# Data for “Oxidative stress is inhibited by plant-based supplements: a quantitative lipidomic analysis of antioxidant activity and lipid compositional change”

**DOI:** 10.1016/j.dib.2022.108879

**Published:** 2023-01-05

**Authors:** Julia Bahja, Nicolas A. Stewart, Marcus K. Dymond

**Affiliations:** Centre for Stress and Age-Related Disease, University of Brighton, BN2 4GL

**Keywords:** Oxidative stress, Membrane curvature, Antioxidant study, Grape seed extract, Pine bark extract, Milk thistle extract, Hawthorn extract, Turmeric extract

## Abstract

Raw data obtained by ultra-high pressure liquid chromatography–mass spectrometry, and processed lipid compositional data are presented alongside detailed methodology. Data were obtained as bovine liver lipid extract oxidizes, initiated by 2,2′-Azobis(2-amidinopropane) dihydrochloride, at 0, 6 and 24 h post initiation. Lipid oxidation data in the presence and absence of some supplements with antioxidant properties was obtained. The supplements used were grape seed extract, pine bark extract, milk thistle extract, hawthorn extract and turmeric extract.

**Specifications Table**

**Table:** 

Subject	Omics: Lipidomics
Specific subject area	Lipidomics data quantifying the oxidation of bovine liver lipids in the presence of supplements with possible antioxidant properties
Type of data	Raw mzXML files Tables
How the data were acquired	Data were acquired using ultra-high performance liquid chromatography (Ultimate 3000, ThermoScientific) coupled with mass spectrometry (hybrid quadrupole Orbitrap mass spectrometer, Q Exactive, ThermoScientific). Reversed phase chromatography (ACQUITY UPLC HSS T3 Column, 100Å, 1.8 µm, 2.1 mm X 100 mm, Waters Corporation) was used to separate the lipids.
Data format	Raw Analyzed
Description of data collection	Collected from an analysis of raw mass spectroscopy files using Proteowizard, Mzmine and Lipidex software.
Data source location	Mass spectrometry data collected in Brighton, UK.
Data accessibility	Data is provided in this article and RAW MS files have been deposited in Mendeley Data DOI: 10.17632/bxv6zyv2xz.1 https://data.mendeley.com/datasets/bxv6zyv2xz/1
Related research article	**Bahja, J., Stewart, N.A. and Dymond, M.K.,** 2022. Oxidative stress is inhibited by plant-based supplements: a quantitative lipidomic analysis of antioxidant activity and lipid compositional change. *Advances in Redox Research*, p.100054.

## Value of the Data

• These data record the changing lipid profile of a complex lipid mixture as it undergoes oxidation and the effect of 5 plant extract supplements on lipid oxidation. 15 lipid classes and the absolute amounts of 378 individual lipids were quantified.
• Researchers with an interest in oxidative stress and antioxidant plant extracts will benefit from these data.
• Analyzed data can be used to parameterize mechanistic models of lipid oxidation and inhibitors of lipid oxidation.
• Raw data can be reanalyzed in the future as the field of oxidative lipidomics develops.
• The methodology developed can be used to quantify the antioxidant effects of other compounds.

## 1 Objective

This dataset provides raw HPLC-MS/MS data [1] and quantitative lipid amounts of a targeted lipidomic analysis, which revealed 378 individual lipids and 15 lipid classes in a bovine liver lipid mixture undergoing oxidative stress. Numerical values of the PC: PE ratio and double bond index (DBI) are also provided. These data support of our recent publication [2] where the inhibitory effects of five plant-based supplements were investigated. This data-in-brief article adds to the existing publication by providing the scientific community with both the raw HPLC- MS/MS data and the processed numerical lipidomics data, used to generate the plots in our publication. Making this data freely available facilitates subsequent reanalysis.

## 2 Data Description

Data presented are the absolute amounts of the individual quantified lipids and their chemical identities obtained as a bovine liver lipid extract is oxidized using the peroxidation agent 2,2′-Azobis(2-amidinopropane) dihydrochloride (AAPH). The antioxidant effect of five different plant extract supplements formed the basis of the experiment, which contrasts lipid oxidation in the presence of these to a control, no supplement, dataset. These data support a recent publication [2] where the possible antioxidant properties of supplements derived from plant extracts were measured using a targeted lipidomics approach. The motivation for the research was to understand how oxidative stress impacts membrane lipid composition [3], collective membrane physical properties [4] and any ameliorating properties of dietary supplements in this context. Using lipidomics with complex lipid mixtures allows the isolated effects of lipid oxidation to be elucidated away from the lipid homeostatic responses of cells, which is particularly interesting since lipids are associated with many cellular processes and organelles through lipid protein or lipid DNA interactions [3,^5^,6].

**Fig. 1:**
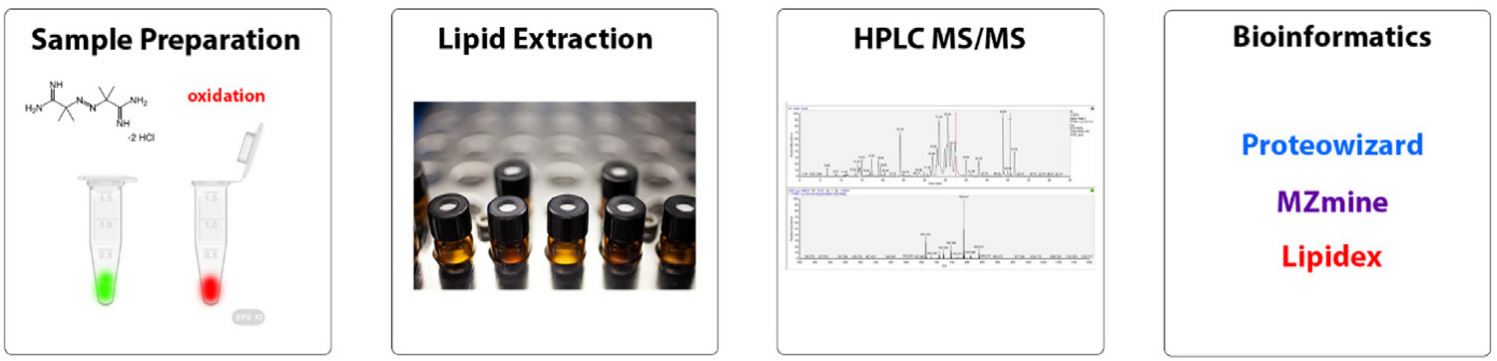
schematic representation of the experimental and data processing steps.

Raw mass spectrometry files have been deposited at Mendeley Data [1]. The data presented in tables herein are the analyzed output of a bioinformatic association of chromatographic features obtained using HPLC/MS to a library of lipid molecules and their fragmentation pathways. Proteowizard [7], Mzmine [8,9] and Lipidex [10] software were used process the raw files, identify chromatographic features and make lipid identifications. This process led to the identification of 15 lipid classes. Fig. 1 shows a general scheme for the experimental process, data collection, bioinformatic analysis and data treatment.

Data for the absolute amounts of individual lipid species obtained (arithmetic mean ± standard deviation) are provided as supporting information. Table S1 shows the total absolute amount (nmoles per 10 µL of oxidation assay) for each lipid class identified (i.e. sum of all individual lipid species in the class). Lipid classes identified were phosphatidylcholine (PC), phosphatidylethanolamine (PE), phosphatidylinositol (PI), phosphatidylglycerol (PG), lysophosphatidylcholine (LysoPC), lysophosphatidylethanolamine (LysoPE), hydroxyphosphatidylcholine (PC(OH)), hydroxyphosphatidylethanolamine (PE(OH)), plasmenylphosphatidylethanolamine (Plasmenyl-PE), plasmanylphosphatidylethanolamine (Plasmanyl-PE), plasmenylphosphatidylcholine (Plasmenyl-PC), plasmanylphosphatidylcholine (Plasmanyl-PC), sphingomyelin (SM), diacylglycerol (DG) and triacylglycerol (TG). Notation of lipids in the accompanying tables uses the Lipidmaps recommendation [11]. Data were obtained at 0, 6 and 24 h in the absence of supplement (i.e. no supplement (NS)) and in the presence of supplements derived from grape seed (GS), hawthorn (HA), pine bark (PB), milk thistle (ML) and turmeric (TU) extracts.

Tables S2 to S16 show the individual lipid species identified and quantified using the methodology. These are DG (Table S2), LysoPC (Table S3), LysoPE (Table S4), PC (Table S5), PC(OH) (Table S6), PE (Table S7), PE(OH) (Table S8), PG (Table S9), PI (Table S10), Plasmanyl-PC (Table S11), Plasmanyl-PE (Table S12), Plasmenyl-PC (Table S13), Plasmenyl-PE (Table S14), SM (Table S15) and TG (Table S16). Table 1 shows two widely used metrics for studying membrane stability these are the PC: PE ratio and the double bond index (DBI), calculated using the data obtained.

**Table 1:** PC: PE ratio and double bond index of oxidizing lipid mixtures in the absence and presence of lipid mixtures. PC:PE_1_ ratio is determined from the total quantified PC and PE lipids. The PC: PE_2_ ratio is calculated as a sum of the lipids (PC, PC(OH), plasmanyl-PC and plasmenyl-PC): (PE, PE(OH), plasmanyl-PE and plasmenyl-PE). DBI is calculated using the method described [12] .

	NS 0	NS 6	NS 24	GS 0	GS 6	GS 24	HA 0	HA 6	HA 24	ML 0	ML 6	ML 24	PB 0	PB 6	PB 24	TU 0	TU 6	TU 24
**PC: PE_1_**	2.9 ± 0.2	5.8 ± 0.6	15.9 ± 0.5	2.9 ± 0.1	4.2 ± 0.1	7.3 ± 0.3	2.9 ± 0.1	4.8 ± 0.4	9.8 ± 0.2	3.1 ± 0.1	4.7 ± 0.3	11.4 ± 0.4	3.2 ± 0.1	4.5 ± 0.6	9.4 ± 0.5	3.1 ± 0.1	5.4 ± 0.3	10.1 ± 0.3
**PC:PE_2_**	2.5 ± 0.1	5.9 ± 0.7	20.2 ± 2.0	2.5 ± 0.1	4.1 ± 0.1	8.0 ± 0.4	2.6 ± 0.1	4.8 ± 0.4	11.1 ± 0.2	2.7 ± 0.1	4.8 ± 0.4	13.7 ± 0.5	2.7 ± 0.1	4.4 ± 0.7	11.4 ± 0.6	2.6 ± 0.1	5.4 ± 0.3	12.8 ± 0.8
**DBI**	82.6 ± 8.2	26.0 ± 5.4	6.3 ± 1.5	82.3 ± 2.5	38.3 ± 3.8	17.5 ± 3.4	79.2 ± 3.4	34.8 ± 3.5	10.6 ± 1.0	81.6 ± 5.0	30.0 ± 8.0	8.6 ± 1.8	84.9 ± 6.2	36.9 ± 7.2	9.9 ± 1.2	84.8 ± 5.3	32.3 ± 2.6	9.1 ± 1.1

## 3 Experimental Design, Materials and Methods

The following materials and methods were used throughout the study as described in full in our recent publication [2], a brief summary of the aspects key to using the data presented follows.

Bovine polar lipid extract (25 mg/mL, Avanti Polar Lipids), Best Naturals Grape Seed Extract 400 mg Tablets (Best Nutritionals LLC, NJ, US), Milk Thistle 3500 mg Tablets High Strength Silymarin (Natural Foundation Supplements, supplied by HD Supplements ltd. Oxford, UK), Super Strength Hawthorn Berry Extract Capsules (100% Natural Co., supplied by Premium Leisure Distribution, Bristol, UK). Simply Pure Organic Turmeric Capsules x 90, 600 mg, (Simply Pure Ltd, Norfolk, UK), Pine Bark Extract 400 mg, 95% Proanthocyanidins (Horbaach Nutritionals, supplied by Piping Rock UK Limited, London, UK). Water (Hypergrade for LC-MS, LiChrosolv®, MerckKGaA), acetonitrile (MSsuprasolve®, Sigma Aldrich), methanol (HPLC grade ≥ 99.9 %), chloroform (stabilsed with amylenes, ≥ 99.5%), isopropanol (Optima™LC/MS Grade, Fisher Scientific), ammonium formate (99.995%, Sigma Aldrich), Trizma (Sigma Aldrich) and 2,2′-Azobis(2-methylpropionamidine) dihydrochloride (Sigma Aldrich).

### 3.1 Lipidomic Data Acquisition by High Performance Liquid Chromatography-Mass Spectrometry (HPLC- MS)

HPLC-MS) was performed on a hybrid quadrupole Orbitrap mass spectrometer (Q Exactive, ThermoScientific) coupled to an ultra-high-performance LC system (Ultimate 3000, ThermoScientific). Lipids were separated using reversed phase chromatography (ACQUITY UPLC HSS T3 Column (100Å, 1.8 µm, 2.1 mm X 100 mm, Waters Corporation) with a solvent gradient consisting of solvent A; (60:40 v/v); water: acetonitrile and 10 mM ammonium formate and solvent B; (90:10) isopropanol: acetonitrile and 10 mM ammonium formate at a flow rate of 200 µL/min. Full details of the method have been published previously [2].

### 3.2 Bioinformatic Association of Chromatographic Features with Lipid Identities

Thermofisher raw format (.raw) file were converted to mzXML files using MSConvert (Proteowizard) [7], using 64 bit binary encoding precision, write index and TPP compatibility settings. Filtering was performed using the peak picking ‘Vendor’ algorithm at MS level 1. Lipid identities were assigned using Lipidex software [10], using the Lipidex_HCD_Formic, Lipidex_HCD_Hydroxy, Lipidex_HCD_Plants, Lipidex_HCD_ULCFA and Lipidex_Splash_ISTD_Formic spectral libraries, using the spectral searching parameters specified previously [2]. HPLC alignment files were generated using MZMine [8], whereby Thermofisher raw format (.raw) files were imported to MZMine and features were detected using the settings identified in our previous publication [2].

### 3.3 Lipid Analysis and Quantification

Lipid quantifications were performed relative to the isotopic standard spiked in at the Bligh-Dyer extraction stage (SPLASH Lipidomix, Avanti Polar Lipids). PE, PE(OH), plasmanyl-PE and plasmenyl-PE species were quantified to the PE standard 15:0-18:1(d7) PE (5.3 µg/mL), PC, PC(OH), plasmanyl-PC and plasmenyl-PC were quantified to the PC standard 15:0-18:1(d7) PC (150.6 µg/mL). DG, TAG, PI, PG and SM lipids were quantified relative to peak areas corresponding to 15:0-18:1(d7) DG (8.8 µg/mL), 15:0-18:1(d7)-15:0 TAG (52.8 µg/mL), 15:0-18:1(d7) PI (8.5 µg/mL), 15:0-18:1(d7) PG (26.7 µg/mL) and d18:1-18:1(d9) SM (29.6 µg/mL), respectively. Lyso PE and lyso PC were quantified relative to peak areas corresponding to 18:1(d7) lyso PE (4.9 µg/mL) and 18:1(d7) lyso PC 23.8 µg/mL). All data are expressed as the mean average plus/ minus the standard deviation of 3 different samples (n = 3).

### 3.4 *Experimental Design:* Lipid *Oxidation Assays*

Liposomes were prepared in amber HPLC vials from 40 µL of bovine polar lipid extract and 10 µL of plant extract solution (1 mg/mL in methanol). After drying under nitrogen, water (200 µL) was added, and samples were vortexed to mix and lyophilized overnight. Dry lipid films were hydrated with buffer (169 µL, 0.1 M Trizma, pH 7.2, Sigma Aldrich) and resuspended by vortexing (10 min) and rested (20 min) at room temperature. After sonication (20 min) and resting (20 min) 4 freeze thaw cycles were performed. Before use in the oxidation assay liposomes were equilibrated (60 min) at room temperature. No supplement controls were prepared in the same way, omitting the addition of the plant extract solutions at the first step.

Oxidation assays were initiated by mixing equal volumes (typically 169 µL) of liposome solution and AAPH solution (15 mM, in Trizma buffer, 0.1 M, pH 7.2), incubating for 0, 6 and 24 h in a water bath (37°C). Reactions were terminated by freezing and after thawing 10 µL of samples were removed and lipids were extracted using the Bligh Dyer protocol [13] as described previously [2].

## CRediT Author Statement

**Julia Bahja:** Conceptualization, Methodology, Investigation, Writing – review & editing; **Nicolas A. Stewart:** Data Curation, Methodology, Investigation, Writing – review & editing; **Marcus K. Dymond:** Conceptualization, Data curation, Formal analysis, Investigation, Methodology, Writing – original draft, Writing – review & editing, Visualization, Supervision, Project administration.

## Ethical Statements

The authors confirm that this research meets all the ethical criteria for publication in Data in Brief. This research does not involve studies with animals or humans and does not require a detailed ethical statement.

## Declaration of Competing Interest

The authors declare that they have no known competing financial interests or personal relationships that could have appeared to influence the work reported in this paper.

## Data Availability

Raw HPLC-MS/MS data for ``Oxidative stress is inhibited by plant-based supplements: a quantitative lipidomic analysis of antioxidant activity and lipid compositional change'' (Original data) (Mendeley Data).

## Appendix Supplementary materials

Image, application 1
